# TP73 is a credible biomarker for predicting clinical progression and prognosis in cervical cancer patients

**DOI:** 10.1042/BSR20190095

**Published:** 2019-08-05

**Authors:** Hui Ye, Xia Guo

**Affiliations:** 1 Department of Obstetrics, Baoji Hospital of Traditional Chinese Medicine, Baoji City, Shaanxi Province 721001, China; 2 Department of Obstetrics and Gynezcology, Hanjiang Hospital Affiliated to Xi’an Medical College, Hanzhong City, Shaanxi Province 723003, China

**Keywords:** biomarker, cervical cancer, immunohistochemistry, TP73

## Abstract

Tumor protein p73 (TP73) has been reported to be dysregulated in various types of human cancer and associated with clinical progression and outcome. Owing to the lack of reports on the correlation between TP73 protein expression and clinicopathologic features of cervical cancer, the aim of our research was to explore the clinical and prognostic significance of TP73 protein expression in cervical cancer patients. In our study, TP73 protein expression was detected by immunochemistry in 118 paraffin-embedded cervical cancer tissue specimens and 40 paraffin-embedded normal cervical epithelium tissue specimens. In the results, we found cervical cancer tissues exhibited high TP73 expression in comparison with normal cervical epithelium tissues, which was consistent with the expression status of TP73 in The Cancer Genome Atlas (TCGA) database. Furthermore, we analyzed the relationships between TP73 expression and clinicopathologic features through using the chi-square test or Fisher’s exact test, and found high expression of TP73 was markedly associated with early clinical stage, less lymph node metastasis, absent distant metastasis, squamous cell carcinoma and favorable histological grade. The Kaplan–Meier method and log-rank test were performed based on the expression level of TP73 in a cervical cancer cohort from the TCGA database, and showed that TP73 expression was positively correlated with overall survival time in cervical cancer patients. Moreover, univariate and multivariate Cox proportional hazards regression model indicated that high TP73 expression was identified as an independent factor for predicting favorable overall survival in cervical cancer patients. In conclusion, TP73 expression is increased in cervical cancer tissues and cells, and acts as a credible biomarker for predicting favorable overall survival in cervical cancer patients.

## Introduction

Cervical cancer is the second common gynecological tumor worldwide with an estimated 569847 newly diagnosed cases in 2018 [1]. Despite the fact that cervical cancer incidence and mortality rates reportedly have declined in most countries, it remained the fourth leading cause of deaths among women worldwide accounting for over 300000 deaths in 2018 [1]. The decreasing incidence and mortality of cervical cancer is mainly attributed to introduction of vaccines and improvement of disease screening [2,3]. Regrettably, there is still lack of novel treatment for cervical cancer patients. Surgery, radiotherapy and chemotherapy are still the major treatment strategies [4]. Therefore, more novel potential prognostic biomarkers and therapeutic targets should be identified for improving the prognosis of cervical cancer patients.

Tumor protein p73 (TP73) is a member of the p53 tumor suppressor protein family, which has been reported to be dysregulated in various types of human cancer and associated with cancer patients’ prognosis [5,6]. In cervical cancer, TP73 staining was originally observed in the basal and parabasal layers of cervical epithelium, high-grade intraepithelial neoplasia and squamous cell carcinoma [7]. Afterward, TP73 was suggested to be overexpressed in cervical cancer tissues compared with normal cervical tissues [8]. Moreover, a microdissection assay was performed in cervical cancer tissues and adjacent normal tissues, and showed high levels of TP73 in cervical cancer tissues [9]. Besides, DNA methylation and genetic mutations were found in TP73 gene during cervical cancer carcinogenesis [10–13]. Due to lack of reports about the correlation between TP73 protein expression and clinicopathologic features of cervical cancer, the aim of our research was to explore the clinical and prognostic significance of TP73 protein expression in cervical cancer patients.

## Materials and methods

### Tumor specimens and clinical data collection

A total of 118 paraffin-embedded cervical cancer tissue specimens and 40 paraffin-embedded normal cervical epithelium tissue specimens were collected from Baoji Hospital of Traditional Chinese Medicine or Hanjiang Hospital Affiliated to Xi’an Medical College with a standard interviewer-administered questionnaire. Age, clinical stage, tumor size, lymph node metastasis, distant metastasis, histological type, histological grade and clinical outcome were included in the questionnaire. The pathological diagnosis of each tissue specimen was confirmed by at least two pathologists. None of the patients had received radiotherapy or chemotherapy prior to surgery or biopsy. The systematic therapy was conducted based on the National Comprehensive Cancer Network (NCCN) guideline for cervical cancer.

### The Cancer Genome Atlas database analysis

The TP73 expression profiles in cervical cancer tissues (*n*=306) and normal cervical tissues (*n*=13) were obtained from The Cancer Genome Atlas (TCGA) database (https://tcga-data.nci.nih.gov). The prognostic value of TP73 was evaluated in cervical cancer cohort (*n*=291) from TCGA database through using Kaplan–Meier method and log-rank test.

### Immunohistochemical analysis

Cervical cancer tissue specimens embedded in paraffin were cut into 3- to 5-μm serial sections and fixed on to the slides. Then, sections were deparaffinized in xylene twice for 10 min, rehydrated through graded ethanol to distilled water. After conducting antigen retrieval using a microwave for 5 min at 95°C, endogenous peroxidase activity and non-specific binding activity were blocked with 3% hydrogen peroxide and 5% nonfat dried milk, respectively. Subsequently, the sections were incubated with anti-human TP73 antibody (1:100 dilution; Abcam, MA, U.S.A.) overnight at 4°C in a humidified chamber. The primary antibody was replaced by immunoglobulin for the negative control. The next day, sections were incubated with horseradish peroxidase-labeled anti-goat IgG secondary antibody (ZSGB-BIO, Beijing, China) at room temperature for 30 min. The DAB (3,3-diaminobenzidine) staining system was used to display the target protein.

### Evaluation of staining

The immunohistochemical results were estimated semi-quantitatively by calculating the percentage of positive cells based on previous study [8], and were independently estimated by at least two pathologists who were blinded to the clinical data. The nuclear positivity of TP73 staining was assessed quantitatively in ten random fields with original magnification ×400, and the cytoplasmic staining of TP73 was excluded as TP73 is a nuclear protein. Cervical cancer tissue specimens with positive staining in more than 50% of cancer cells were considered as high-expression of TP73, otherwise as low-expression of TP73.

### Statistical analysis

Statistical analyses were conducted by using SPSS version 17.0 (Chicago, IL, U.S.A.). Relationships between TP73 expression and clinicopathologic features were examined through using the chi-square test or Fisher’s exact test. The overall survival curve was drawn by Kaplan–Meier method, and the significant differences of survival curves were estimated by the log-rank test. Univariate and multivariate Cox proportional hazards regression models were applied to assess the independent prognostic factor for overall survival of cervical cancer patients. A *P*-value of less than 0.05 was considered statistically significant.

## Results

### The expression status of TP73 in cervical cancer

**Figure 1:**
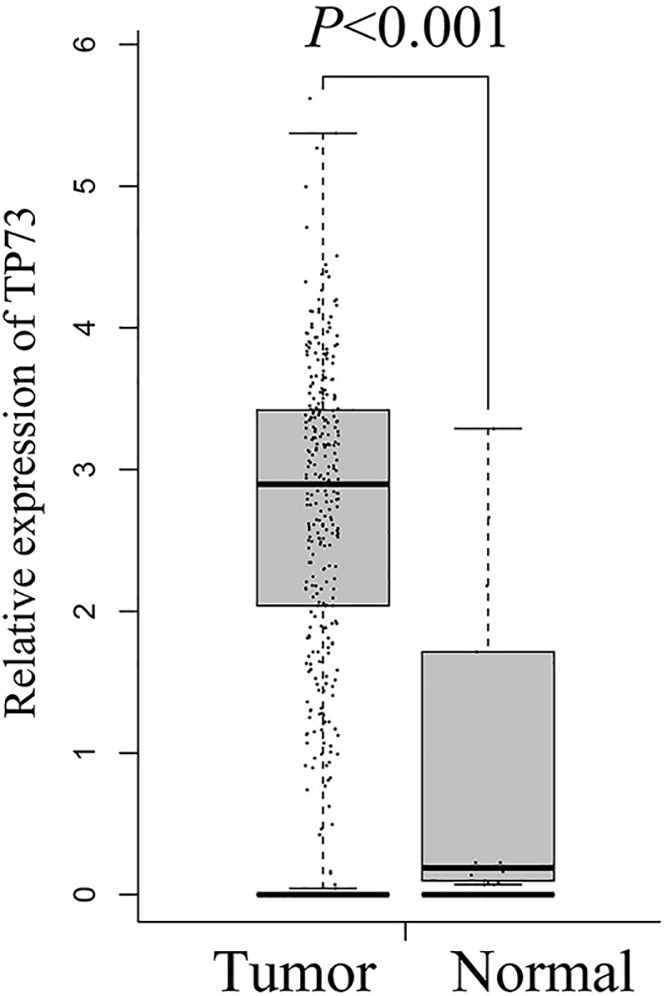
The expression status of TP73 in cervical cancer TP73 expression was estimated in cervical cancer tissues and normal cervical epithelium tissues in TCGA database.

**Figure 2:**
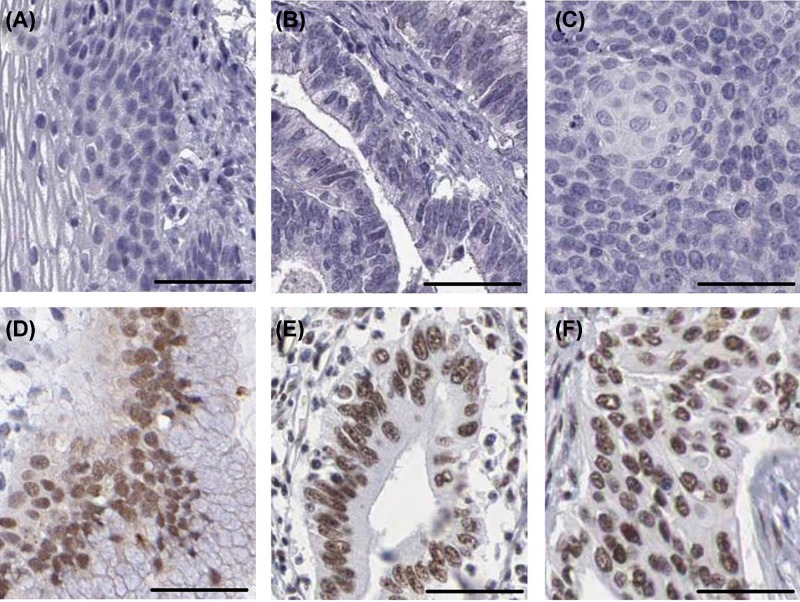
Immunohistochemical staining of cervical cancer (**A**) Negative expression of TP73 in normal cervical epithelium tissues. (**B**) Negative expression of TP73 in cervical adenocarcinoma tissues. (**C**) Negative expression of TP73 in cervical squamous cell carcinoma tissues. (**D**) Positive expression of TP73 in normal cervical epithelium tissues. (**E**) Positive expression of TP73 in cervical adenocarcinoma tissues. (**F**) Positive expression of TP73 in cervical squamous cell carcinoma tissues (scale: 50 μm).

In order to explore the expression status of TP73 in cervical cancer, we first observed TP73 expression in cervical cancer tissues and normal cervical epithelium tissues using the TCGA database. In the TCGA database, the TP73 expression was obviously up-regulated in cervical cancer tissues compared with normal cervical epithelium tissues (*P*<0.001, Figure 1). Moreover, we further performed immunohistochemical analysis to assess TP73 protein expression in cervical cancer tissues and normal cervical epithelium tissues (Figure 2A–F). TP73 was mainly expressed in the nucleus. In normal cervical epithelium tissues, 50.8% (60/118) of samples exhibited high TP73 expression. While in the cervical cancer tissues, high-expression of TP73 was observed in 12.5% (5/40) tumor samples. The statistical result suggested that there was significant difference in TP73 expression between cervical cancer tissues compared with normal cervical epithelium tissues (*P*<0.001, Table 1), which was consistent with the results of TCGA database.

**Table 1:** TP73 protein expression in cervical cancer tissues and normal cervical epithelium tissues

Group	*n*	TP73 protein expression		*P*
		Low (%)	High (%)	
Normal	40	35 (87.5)	5 (12.5)	<0.001
Tumor	118	58 (49.2)	60 (50.8)	

### The clinical significance of TP73 in cervical cancer

The correlations between TP73 expression and clinicopathological features in cervical cancer were further estimated to explore the clinical significance of TP73. Statistical analysis showed that high expression of TP73 was markedly associated with clinical stage (I–IIA vs. IIB–IV, *P*<0.001, Table 2), lymph node metastasis (Absent vs. Present, *P*<0.001, Table 2), distant metastasis (Absent vs. Present, *P*=0.004, Table 2), histological type (adenocarcinoma vs. squamous cell carcinoma, *P*=0.001, Table 2), and histological grade (well vs. moderately/poorly, *P*<0.001, Table 2). However, we did not find statistical correlations of TP73 expression with patients age (*P*=0.483, Table 2), tumor size (*P*=0.365, Table 2) and HPV infection (*P*=0.602, Table 2).

**Table 2:** Associations between TP73 protein expression and clinicopathological characteristics in cervical cancer patients

Characteristics	*n*	TP73 protein expression		*P*
		Low (%)	High (%)	
Age (y)				
≤50	53	23 (43.4)	30 (56.6)	0.483
>50	65	35 (50.0)	30 (50.0)	
Clinical stage				
I–IIA	49	14 (28.6)	35 (71.4)	<0.001
IIB–IV	69	44 (63.8)	25 (36.2)	
Tumor size (cm)				
≤4	66	30 (45.5)	36 (54.5)	0.365
>4	52	28 (53.8)	24 (46.2)	
Lymph node metastasis				
Absent	65	19 (29.2)	46 (70.8)	<0.001
Present	53	39 (73.6)	14 (26.4)	
Distant metastasis				
Absent	107	48 (44.9)	59 (55.1)	0.004
Present	11	10 (90.9)	1 (9.1)	
HPV				
Absent	38	20 (52.6)	18 (47.4)	0.602
Present	80	38 (47.5)	42 (52.5)	
Histological type				
Adenocarcinoma	16	14 (87.5)	2 (12.5)	0.001
Squamous cell carcinoma	102	44 (43.1)	58 (56.9)	
Histological grade				
Well	50	13 (26.0)	37 (74.0)	<0.001
Moderately/poorly	68	45 (66.2)	23 (33.8)	

### The prognostic significance of TP73 in cervical cancer

**Figure 3:**
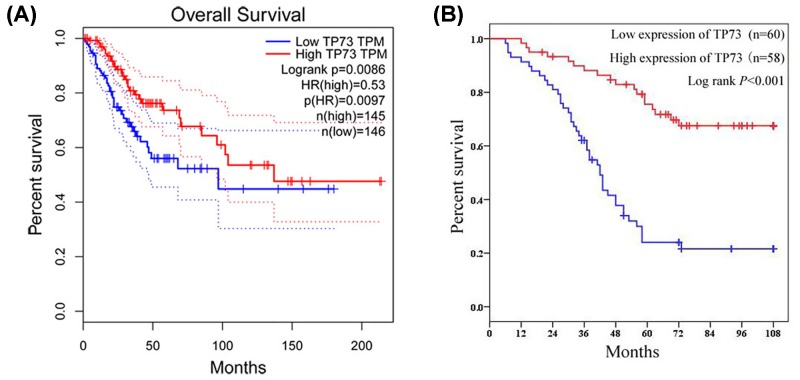
The prognostic significance of TP73 in cervical cancer Kaplan–Meier method and log-rank test were performed based on expression level of TP73 in cervical cancer cohort from TCGA database (**A**) and our study (**B**).

We further evaluated the association between TP73 expression and overall survival of cervical cancer patients for investigating the prognostic significance of TP73. First, the Kaplan–Meier method and log-rank test were performed based on expression level of TP73 in cervical cancer cohort from the TCGA database, and showed that TP73 expression was positively correlated with overall survival time in cervical cancer patients (*P*=0.009, Figure 3A). The overall survival curve of our study also showed cervical cancer patients with high expression of TP73 had better clinical outcome than those with low expression of TP73 (*P*<0.001, Figure 3B). In addition, univariate and multivariate Cox proportional hazards regression models were applied to assess the independent prognostic factor for overall survival of cervical cancer patients. We observed clinical stage (I–IIA vs. IIB–IV, *P*<0.001, Table 3), lymph node metastasis (Absent vs. Present, *P*<0.001, Table 3), distant metastasis (Absent vs. Present, *P*=0.001, Table 3), histological type (adenocarcinoma vs. squamous cell carcinoma, *P*<0.001, Table 3), histological grade (well vs. moderately/poorly, *P*<0.001, Table 3) and TP73 expression (Low vs. High, *P*<0.001, Table 3) were prognostic factors for overall survival in cervical cancer patients in univariate Cox proportional hazards regression model. Furthermore, the result of multivariate Cox proportional hazards regression model suggested that high TP73 expression was an independent factor for predicting unfavorable overall survival in cervical cancer patients (*P*=0.039, Table 3).

**Table 3:** Univariate and multivariate Cox regression analyses of overall survival in cervical cancer patients

Parameter	Univariate analysis			Multivariate analysis		
	*P*	HR	95% CI	*P*	HR	95% CI
Age (y)						
(≤50 vs. >50)	0.097	1.551	0.923–2.606			
Clinical stage						
(I-IIA vs. IIB-IV)	<0.001	2.950	1.669–5.213	0.437	0.667	0.241–1.850
Tumor size (cm)						
(≤4 vs. >4)	0.610	0.877	0.530–1.452			
Lymph node metastasis						
(Absent vs. Present)	<0.001	4.036	2.331–6.987	0.123	2.190	0.809–5.926
Distant metastasis						
(Absent vs. Present)	0.001	3.148	1.607–6.169	0.005	0.188	0.059–0.601
HPV						
(Absent vs. Present)	0.363	1.287	0.747–2.216			
Histological type						
(Adenocarcinoma vs. Squamous cell carcinoma)	<0.001	0.219	0.210–0.400	0.001	0.156	0.054–0.451
Histological grade						
(Well vs. Moderately/Poorly)	<0.001	6.664	3.355–13.238	<0.001	4.133	1.911–8.937
TP73 protein expression						
(Low vs. High)	<0.001	0.240	0.137–0.421	0.039	0.505	0.263–0.967

Abbreviations: HR, hazard ratio; 95% CI, 95% confidence interval.

## Discussion

TP73 is a member of the TP53 family and has multiform isoforms with different functions and clinical significance in human cancers [14]. Since immunohistochemistry is the most common method in pathologic diagnosis and clinical practice, we conducted immunohistochemical staining to detect TP73 expression, and analyzed the clinical and prognostic values of TP73 in cervical cancer. In our study, we found that cervical cancer tissues exhibited high TP73 expression in comparison with normal cervical epithelium tissues, which was consistent with the expression status of TP73 in TCGA database. Similarly, Liu et al. [8] also reported that TP73 was overexpressed in cervical cancer tissues compared with normal cervical tissues. In addition, high expression of TP73 was suggested in several kinds of human tumors such as breast cancer [15,16], esophageal cancer [17], thymic carcinoma [18], extrahepatic bile duct carcinoma [19], cholangiocellular carcinoma [20], hepatocellular carcinoma [21,22], gastric cancer [23,24], colorectal cancer [25–27], ovarian cancer [28], laryngeal cancer [29], parotid gland carcinoma [30], cutaneous melanoma [31] and retinoblastoma [32]. However, low TP73 expression was observed in bladder cancer and head and neck squamous cell carcinoma, compared to corresponding normal tissues [33,34]. Besides, there was no significant difference in TP73 expression between malignant salivary gland tumors and benign salivary gland lesions [35]. Generally, immunohistochemical staining TP73 was suggested to be high levels in most types of human cancer.

Relationships between TP73 expression and clinicopathologic features have not been reported in cervical cancer patients. Thus, we further analyzed the clinical significance of TP73 expression in cervical cancer patients, and found high expression of TP73 was markedly associated with early clinical stage, less lymph node metastasis, absent distant metastasis, squamous cell carcinoma and favorable histological grade. In bladder cancer, Puig et al. [33] found high TP73 expression was often observed in invasive tumors than in superficial lesions. Moreover, Chen et al. [36] showed esophageal squamous cell carcinoma patients with favorable histopathologic classification had higher levels of TP73 expression than those with unfavorable histopathologic classification. In addition, Ito et al. [37] suggested high TP73 expression was negatively correlated with tumor size, lymph node metastasis and Ki-67 labeling index in patients with pancreatic adenocarcinoma. On the contrary, several studies indicated that high TP73 expression was associated with clinical progression in human cancers. In breast cancer, high TP73 expression was connected to more metastatic lymph nodes, vascular invasion, and advanced pathological stage [15,16]. Moreover, Hong et al. [19] found TP73 overexpression was associated with deeper tumor invasion in patients with extrahepatic bile duct carcinoma. In retinoblastoma patients, Adithi et al. [32] showed patients with high risk tumor had higher TP73 expression than those with low risk tumor. Zhang et al. [38] demonstrated metastatic melanoma tissues exhibited high TP73 expression in comparison with primary melanoma tissues. However, there was no statistical association between TP73 expression and clinicopathologic features in hepatocellular carcinoma [39], colorectal cancer [25,40,41] and ovarian cancer [42]. Generally, the clinical significance of TP73 expression had obvious difference depending on the type of human cancers.

Most studies have shown that positive immunohistochemical staining TP73 was associated with worse clinical outcome in extrahepatic bile duct carcinoma [19], cholangiocellular carcinoma [20], hepatocellular carcinoma [39,43], colorectal cancer [26,44], ovarian cancer [28] and esophageal squamous cell carcinoma [36]. However, we further evaluated the association between TP73 expression and overall survival of cervical cancer patients in TCGA database, and found that TP73 expression was positively correlated with overall survival time in cervical cancer patients. Furthermore, the overall survival curve of our study also showed cervical cancer patients with high expression of TP73 had better clinical outcome than those with low expression of TP73. In addition, Liu et al. [8] similarly suggested that TP73 overexpression predicted favorable clinical outcome in cervical cancer patients. Furthermore, high TP73 expression was identified as an independent factor for predicting favorable overall survival in cervical cancer patients through univariate and multivariate Cox proportional hazards regression model. Generally, high TP73 expression is a credible biomarker for predicting favorable prognosis in cervical cancer patients.

Our data and Liu et al.’s [8] data showed that TP73 expression was up-regulated in cervical cancer tissues, and served as an independent factor for predicting favorable overall survival in cervical cancer. Yet, due to the limited sample size of patients in these two studies, further research is still needed to confirm these findings and establish the clinical value of TP73 expression as a reliable prognostic predictor for the outcome of cervical cancer patients.

## Conclusion

TP73 expression is up-regulated in cervical cancer tissues, and negatively associated with clinical progression in cervical cancer patients. High TP73 expression is an independent factor for predicting favorable overall survival in cervical cancer.

## Abbreviations

TCGA: The Cancer Genome Atlas
TP73: tumor protein p73
